# RNA-Seq analysis of a Pax3-expressing myoblast clone *in-vitro* and effect of culture surface stiffness on differentiation

**DOI:** 10.1038/s41598-022-06795-3

**Published:** 2022-02-18

**Authors:** Louise Richardson, Dapeng Wang, Ruth Hughes, Colin A. Johnson, Michelle Peckham

**Affiliations:** 1 School of Molecular and Cellular Biology, University of Leeds, Leeds, UK; 2 LeedsOmics, University of Leeds, Leeds, LS2 9JT UK; 3 Wellcome Centre for Human Genetics, University of Oxford, Oxford, OX3 7BN UK; 4 Leeds Institute of Medical Research, Faculty of Medicine and Health, University of Leeds, Leeds, UK

**Keywords:** Muscle stem cells, RNA sequencing

## Abstract

Skeletal muscle satellite cells cultured on soft surfaces (12 kPa) show improved differentiation than cells cultured on stiff surfaces (approximately 100 kPa). To better understand the reasons for this, we performed an RNA-Seq analysis for a single satellite cell clone (C1F) derived from the H2k^b^-tsA58 immortomouse, which differentiates into myotubes under tightly regulated conditions (withdrawal of ɣ-interferon, 37 °C). The largest change in overall gene expression occurred at day 1, as cells switched from proliferation to differentiation. Surprisingly, further analysis showed that proliferating C1F cells express Pax3 and not Pax7, confirmed by immunostaining, yet their subsequent differentiation into myotubes is normal, and enhanced on softer surfaces, as evidenced by significantly higher expression levels of myogenic regulatory factors, sarcomeric genes, enhanced fusion and improved myofibrillogenesis. Levels of mRNA encoding extracellular matrix structural constituents and related genes were consistently upregulated on hard surfaces, suggesting that a consequence of differentiating satellite cells on hard surfaces is that they attempt to manipulate their niche prior to differentiating. This comprehensive RNA-Seq dataset will be a useful resource for understanding Pax3 expressing cells.

## Introduction

Satellite cells are the stem cells of skeletal muscle. They lie underneath the basal lamina of the muscle fibre, and transition from a quiescent to an activated state to repair, regenerate or grow muscle fibres in vivo (reviewed in^1,2^). Following activation, they proliferate to form new daughter satellite cells that replenish the stem cell niche or differentiate into muscle fibres. Satellite cells can be isolated from skeletal muscle and the resulting myoblasts recapitulate the process of muscle differentiation in culture into multinucleated myotubes (reviewed in^3^).

Most studies of cultured myoblasts use the C2C12 muscle cell line, a subclone of C2 cells originally isolated as a spontaneously transformed myoblast cell line^4,5^. Differentiation of C2C12 cells is typically initiated by allowing the cells to become confluent and changing the medium from growth to differentiation medium. However, these cells have some differences from primary satellite cells^3^ including the ability to induce tumour formation if introduced into muscle in vivo^6^.

An alternative to C2C12 cells, is to use a myoblast clone isolated from the H2k^b^-tsA58 immortomouse^7^. These cells contain the temperature sensitive mutant of the SV40 large T-antigen (tsA58) under the control of an inducible promoter (*H-2k*^*b*^). The addition of interferon-ɣ (IFN-ɣ) drives transcription of the tsA58 gene, and at a temperature of 33 °C the T-antigen is stable and promotes cell proliferation. Removing IFN-ɣ stops transcription and increasing the temperature to 37–39 °C leads to degradation of any remaining expressed T-antigen, allowing the myoblasts to exit the cell cycle and differentiate^8^. These cells have proved valuable in the area of myoblast research as they more faithfully recapitulate the behaviour of primary myoblasts, and do not form tumours when transplanted into mice^8^. Moreover, these cells can be derived as single clones, with varying behaviour. For example, one clone (2B4) exhibits stem cell behaviour^9^, while other clones show differences in adult skeletal myosin isoform expression in differentiating myotubes^10^.

The aim of this research was to perform an extensive differential gene expression analysis of a single H2k^b^-tsA58 myoblast clone (C1F) during differentiation, to characterize changes in muscle gene expression. This clone was isolated previously from the H2k^b^-tsA58 immortomouse^8,10^. In addition, we tested how gene expression was affected by culturing these cells on soft surfaces, in which the stiffness matches that of the muscle fibre (approximately 12 kPa) compared to hard (approximately 100 kPa) glass or plastic surfaces. C2C12 cells differentiate optimally on these soft surfaces^11^. Finally, the differential gene analysis revealed that the specific myoblast clone we analysed here (C1F) predominantly expresses Pax3 and not Pax7. Although both Pax3 and Pax7 are important for satellite cell formation, Pax3 positive satellite cells are rarer than Pax7 positive satellite cells (reviewed in^1^), and this has enabled us to explore the differentiation of this rarer type of satellite cell in vitro.

## Results

### Overall gene expression is affected by the culture surface

**Figure 1:**
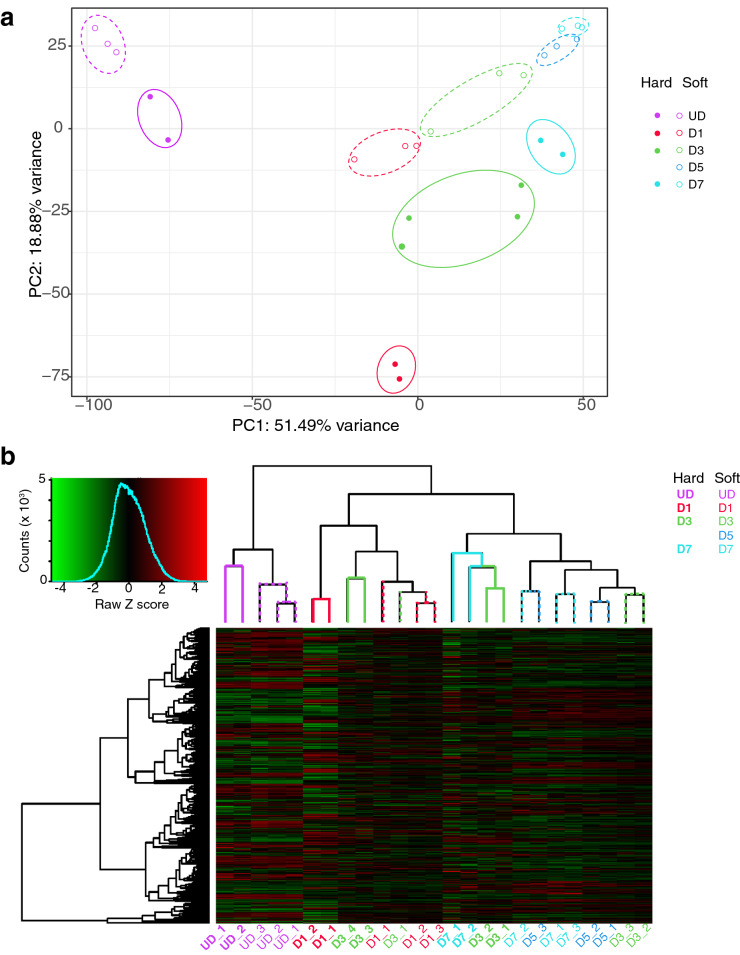
PCA plot and dendrogram for C1F cells differentiating on hard or soft surfaces. (**a**) PCA analysis for each set of samples at each time point. ‘Hard’ represents hard surfaces (approximately 100 kPa stiffness). ‘Soft’ represents soft surfaces (approximately 12 kPa stiffness). At each time point, the values for samples (replicates) on hard surfaces and soft surfaces are shown as individual points. (**b**) Hierarchical clustering of gene expression for cells grown on hard or soft surfaces at different each time point. The branch length at the top of the figures indicates the level of dissimilarity between sample clusters (those with shorter branches have a higher degree of similarity). Each individual branch represents a group of related genes expressed within the corresponding sample (replicate) labelled underneath.

The principal component analysis (PCA) (Fig. 1a) shows that at each day of sampling, the RNA-Seq data for samples from cells cultured on hard surfaces clusters separately to samples from cultures cultured on soft surfaces. This indicates that gene expression patterns within each set of samples are highly correlated and is consistent with the idea that gene expression is affected by the surface on which the cells were grown. Repeats for the same conditions do not completely overlap because of batch-to-batch variation. A hierarchical analysis (Fig. 1b) also shows that samples within each treatment (hard vs soft) cluster together but samples from different treatments are less closely linked. Overall, there is a marked difference in gene expression between cells grown on standard hard plastic surfaces compared to soft (approx. 12 kPa stiffness) polydimethylsiloxane (PDMS) surfaces.

### Comparison of overall gene expression between hard and soft surfaces over time

**Figure 2:**
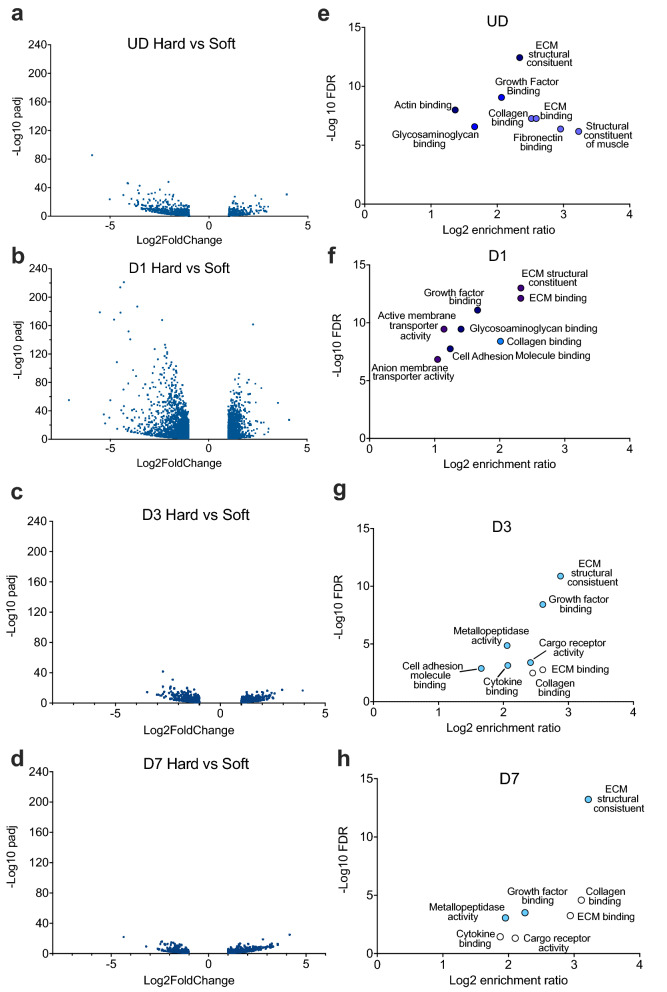
Global comparison of changes in gene expression levels at each time point between C1F cells cultured on hard or soft surfaces. (**a–d**) Shows volcano plots for gene expression where the log_2_fold change is either > 1 or < − 1, and where the padj is < 0.05 (− log10 padj > 1.3). (**e–h**) Shows the overrepresented GO (Gene Ontology) terms with false discovery rate (FDR) < 0.05 from a WebGestalt analysis for differentially-expressed-genes between hard and soft surface samples. The symbol colour for each gene set is approximately related to the number of enriched genes within each gene set from dark blue (> 100) to white (< 20).

We next performed an analysis of the overall variation in gene expression levels between cells grown on hard and soft surfaces at each time point (Fig. 2a–d). A global analysis of the change in expression levels across genes in which the change in expression is significant (_padj_ < 0.05; log_2_FoldChange < − 1 or > 1), showed that the largest difference in gene expression occurred at day 1 (D1) of differentiation. At this time point, the cells switch from proliferation to differentiation, and we expect a large change in gene expression as genes expressed by proliferating cells are switched off and genes that drive differentiation are switched on. As the soft surfaces are expected to promote the switch from proliferation to differentiation, this is consistent with the idea that soft surfaces do promote this switch. Pattern analysis (see methods) of the RNA-Seq data on hard soft surfaces showed a higher number of clusters for changes to gene expression in cells cultured on soft surfaces compared to those cultured on hard surfaces (Supplemental Fig. S1).

A WebGestalt analysis of genes expressed at > twofold higher (log_2_FoldChange > 1) levels on hard than on soft surfaces (p_adj_ < 0.05) revealed that mRNA expression for genes associated with the structural constituent of muscle and those for extracellular matrix (ECM) were higher on hard surfaces in undifferentiated cells (Fig. 2e). Genes encoding ECM structural constituents, ECM binding and growth factor binding were enriched for cells cultured on hard surfaces across the time course (Fig. 2e–h). To explore these changes further we first analysed expression of transcription factor genes associated with myogenesis and genes associated with myofibrillogenesis (formation of muscle sarcomeres).

### Changes in myogenic transcription factor expression between hard and soft surfaces

**Figure 3:**
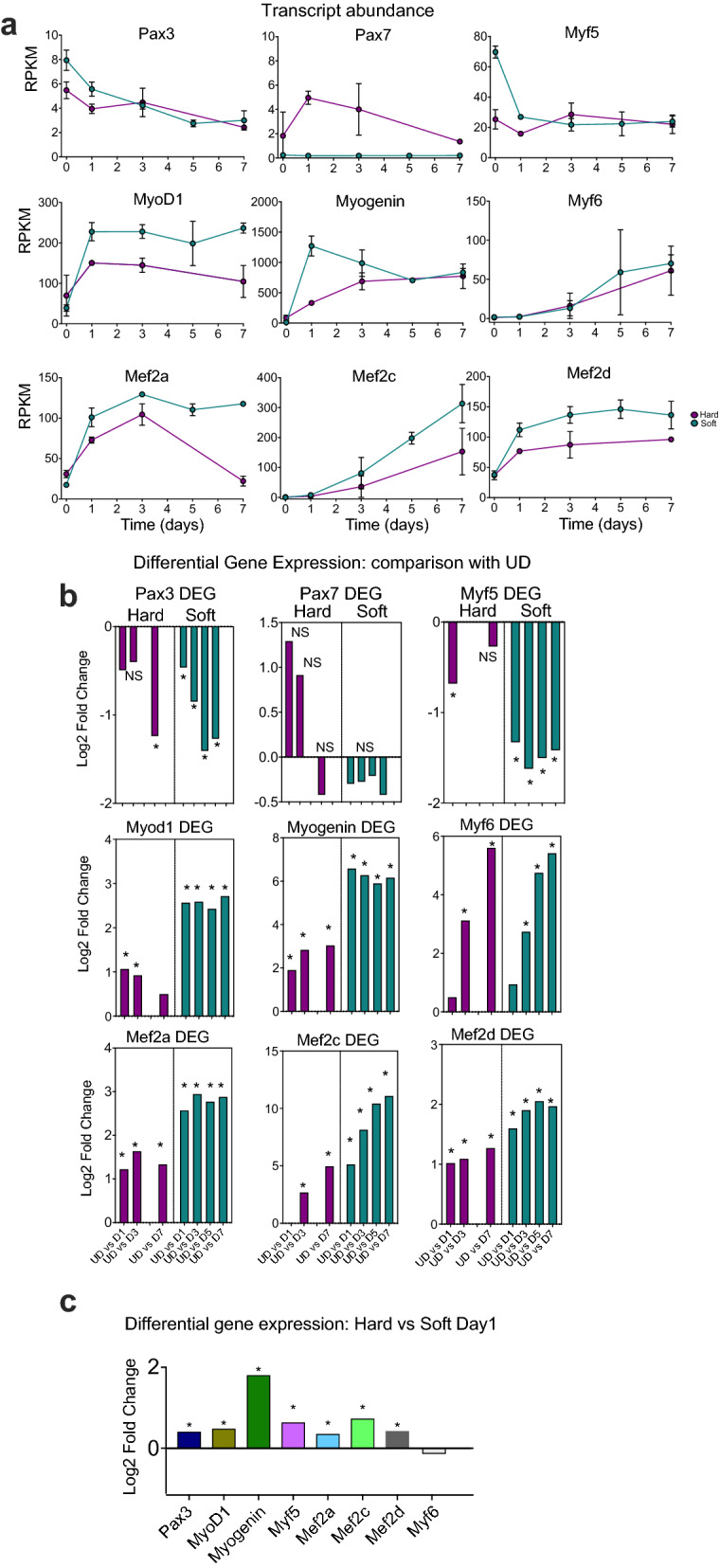
Expression of myogenic factors on hard or soft surfaces in C1F cells. (**a**) RPKM values (reads per kilo base per million mapped reads: transcript abundance) plots for hard (magenta) and soft (green) surfaces over time. Day 0 (D0) represents undifferentiated cells (UD). Days 1–7 represent the time course of differentiation. Error bars show the standard deviation (S.D.). (**b**) Differential gene analysis (DEG) that compares expression for a specific gene on each day of differentiation (D1-7) to undifferentiated cells (UD) for cells cultured on hard and on soft surfaces. *Indicates significant change in log_2_fold expression (p_adj_ value < 0.05). (**c**) Differential gene analysis that compares expression levels of each gene directly for hard versus soft surfaces, at each time point (from UD to D7). *Indicates significant change in log_2_fold expression (p_adj_ value < 0.05).

Quiescent and newly activated satellite cells typically express one or both of two paired box transcription factor genes, Pax3 (paired box 3) and Pax7 (paired box 7). Pax7 is more commonly expressed in satellite cells than Pax3^1^ and muscle cell lines such as C2C12 express Pax7^12^. The RNA-Seq data demonstrated that undifferentiated (UD) C1F cells on soft and hard surfaces express Pax3 and that levels of Pax3 decrease as the cells differentiate, as expected (Fig. 3a). To explore this further, we performed a differential gene expression (DEG) analysis that independently compared mRNA levels of Pax3 at each day (from D1 to D7) to those in undifferentiated cells (UD, at Day 0) (Fig. 3b). This showed that for cells cultured on hard surfaces, Pax3 levels did not significantly decrease compared to UD until D7 (Fig. 3b). In contrast, for cells cultured on soft surfaces, levels of Pax3 were reduced significantly at each day (D1-D7) of differentiation compared to UD cells (Fig. 3b). We then performed a second differential gene expression analysis (DEG) that directly compares Pax3 mRNA levels for cells cultured on hard surfaces, to those cultured on soft surfaces at the same time point (D1) (Fig. 3c). This showed a small but significant decrease in levels of Pax3 on hard compared to soft surfaces (Fig. 3c). This suggests that soft surfaces might promote higher mRNA levels of Pax3.

In contrast, levels of Pax7 mRNA in cells cultured on soft surfaces were very low compared to cells on hard surfaces (Fig. 3a). Expression of Pax7 appears to be higher for cells grown on hard surfaces and appears to increase from UD to D1 and D3 (Fig. 3a,b). However, RPKM (reads per kilobase per million mapped reads) values for Pax7 are low: 50% lower than for Pax3 even on hard surfaces (Fig. 3a). A differential gene analysis showed no significant change in expression between UD cells and D1–7 cells for cells cultured on both types of surfaces (Fig. 3b). Overall, these results suggest that the C1F cell line is unusual in that it predominantly expresses Pax3 rather than Pax7.

Next, we investigated the expression patterns of the four myogenic regulatory factors Myf5 (myogenic factor 5), MyoD (myoblast determination protein 1), myogenin and Myf6 (myogenic factor 6: also known as MRF4), which belong to the helix-loop-helix family of transcription factors^13,14^. The expression of Myf5, MyoD, myogenin and Myf6 is hierarchical, with Myf5 and MyoD being expressed first, followed by myogenin and Mrf4 (Myf6)^13^. Myf5 can also be expressed in quiescent satellite cells and/or throughout differentiation^15^.

Myf5 mRNA levels decreased from UD to D1 and then remained low for cells grown on both soft and hard surfaces (Fig. 3a). A differential gene expression analysis that independently compares mRNA levels at D1-D7 with UD, showed that Myf5 levels decreased significantly at D1 compared to UD on both surfaces (Fig. 3b). MyoD mRNA levels increased at D1 and then remained roughly similar on both surfaces (Fig. 3a). A differential gene expression analysis showed that the levels of MyoD were increased significantly at D1-D3 compared to UD cells on both surfaces and were also increased significantly at D5 and D7 compared to UD on soft surfaces (Fig. 3b). Myogenin levels also increased from UD to D1-7 for cells cultured on both surfaces (Fig. 3a) and the differential gene expression analysis showed a significant increase in levels at D1-D7 compared to UD (Fig. 3b). However, the increase in myogenin level was much lower at D1 on hard compared to soft surfaces (Fig. 3c). Myf6 levels were significantly increased at later time points, from D3 onwards, compared to UD cells (Fig. 3a,b) for cells cultured on both soft and hard surfaces. This later rise is consistent with previous work^15^.

The Mef2 (myocyte enhancer factor 2) family of genes (Mef2a,b,c and d) encode myogenic transcription factors that belong to the MADS family of transcription factors^16^. Mef2c can act synergistically with MyoD and myogenin to promote myogenesis, while Mef2a and d can act synergistically with MyoD, and this is mediated through direct interaction of the two proteins^17^. Mef2c has also been shown to be important for the maintenance of sarcomere integrity, by regulating transcription of the sarcomeric gene myomesin^18^. We found that levels of Mef2a,c and d increased significantly from UD cells to D1-7 cells on both hard and soft surfaces although the magnitude of the log_2_fold change was higher on soft surfaces (Fig. 3a,b).

A further differential gene expression analysis that independently compares mRNA levels for cells cultured on hard surfaces, to those cultured on soft surfaces at the same time point was performed (Fig. 3c). This showed that levels of each of the myogenic factors was significantly higher for cells on soft, compared to hard surfaces, with the exception of Myf6 (MRF4) (Fig. 3c). Strikingly, the increase in myogenin levels between UD and D1 was much higher for cells on soft surfaces compared to those on hard surfaces (Fig. 3c). As myogenin is a master regulator of differentiation, this indicates that the cells on soft surfaces may be differentiating into myotubes at a faster rate than those on hard surfaces.

**Figure 4:**
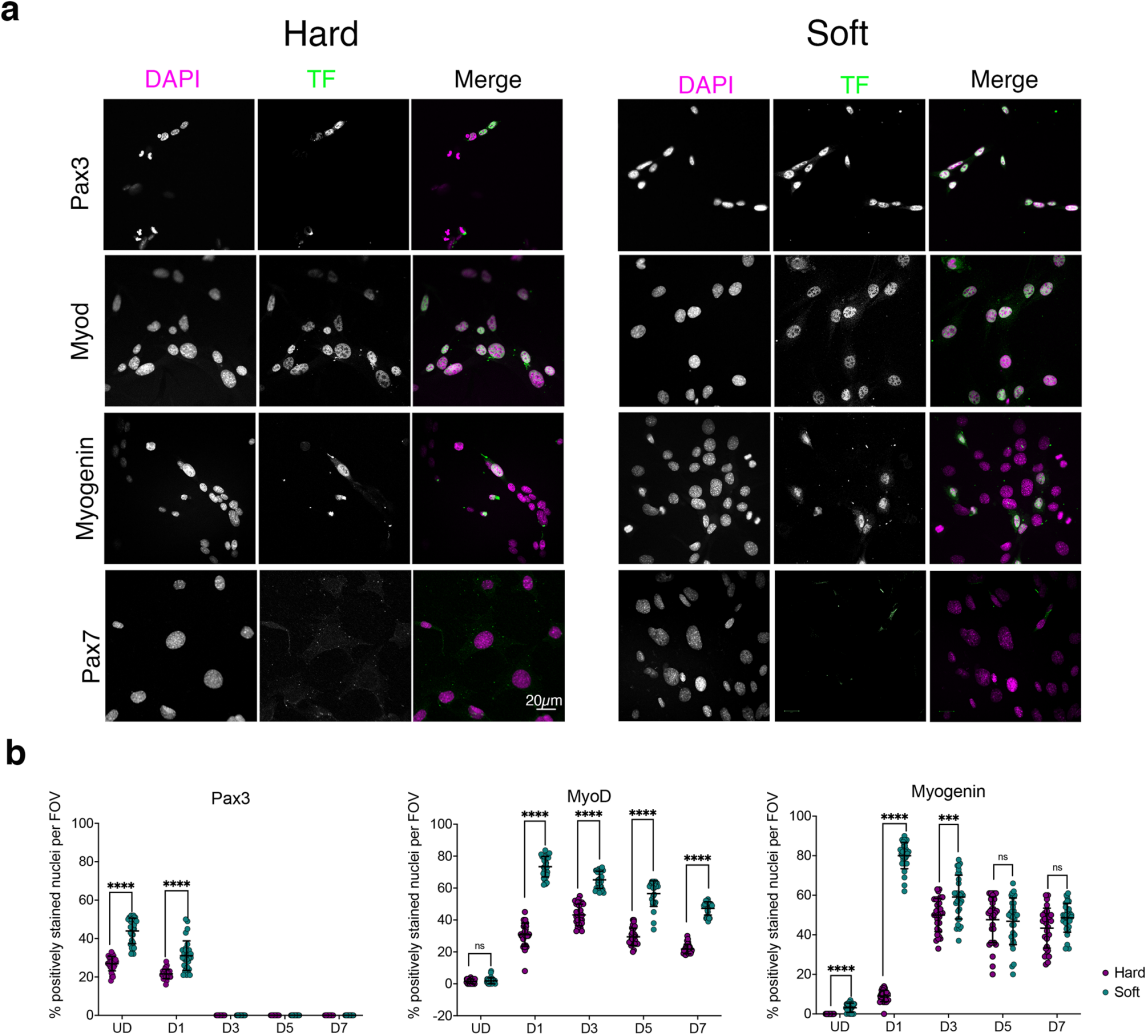
Immunostaining for myogenic factors in C1F cells cultured on hard or soft surfaces. (**a**) Example images of cells stained for DAPI and specific transcription factor, shown in greyscale for each channel, and as magenta and green respectively in the merged image. (**b**) Quantification of the number of nuclei staining positively for each transcription factor (Pax3, MyoD and myogenin) over time on hard (magenta) and soft (green) surfaces. ****p < 0.0001, ***p < 0.001. *ns* non-significant.

To confirm changes in mRNA expression, we analysed C1F cells fixed and stained for a subset of myogenic transcription factors: Pax3, MyoD and myogenin from undifferentiated cells to day 7 of differentiation (Fig. 4a). Numbers of nuclei positive for Pax3 were significantly higher in CIF cells on soft compared to hard surfaces in undifferentiated cells and at D1, and then were undetectable from D3-D7 (Fig. 4b). Numbers of nuclei positive for MyoD increased at D1 and then gradually declined, with numbers significantly higher in cells on soft surfaces from D1-D7 (Fig. 4b). On soft surfaces, numbers of nuclei positive for myogenin increased at D1 and then gradually declined, while on hard surfaces, numbers did not increase until D3 (Fig. 4b). Numbers of nuclei positive for myogenin were significantly higher in cells cultured on soft surfaces compared to hard surfaces at D1 and D3. We additionally stained for Pax7, but no positively stained nuclei were detected, consistent with the RNA-Seq data (Fig. 4a). Overall, these results are in broad agreement with the RNA-Seq analysis and confirm that the C1F cell express Pax3 and not Pax7.

**Figure 5:**
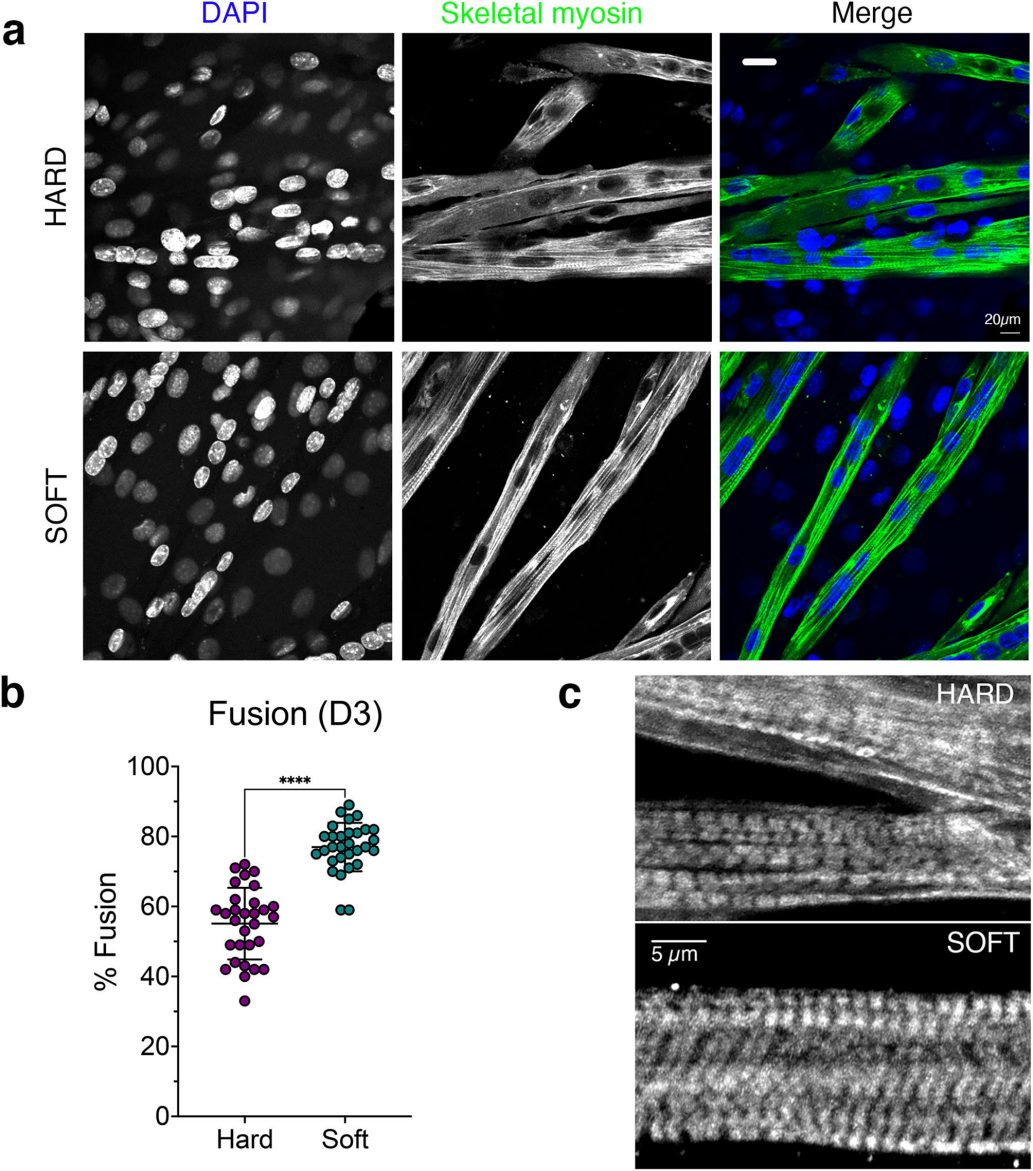
Fusion and differentiation of C1F cells on hard or soft surfaces. (**a**) representative fields of view for differentiating cells stained for nuclei (DAPI) and skeletal myosin, shown in greyscale for each channel, and as blue (DAPI) and green (skeletal myosin) in merged image. (**b**) Fusion index measured for cells on hard (magenta) and soft (green) surfaces at D3. (**c**) Greyscale images of myotubes at day 5 on hard and soft surfaces stained for skeletal myosin, to show sarcomeric organisation.

The rapid increase in myogenin mRNA levels for cells on soft surfaces at D1 further suggested that fusion might be promoted on soft surfaces. To investigate this further, we fixed and stained the cells for skeletal myosin heavy chain at day 3 (D3: Fig. 5a) and quantified fusion on the two types of surfaces (Fig. 5b). The fusion index was indeed increased on soft surfaces (Fig. 5b). This is consistent with the early rise in myogenin expression observed for cells cultured on soft surfaces. Imaging the organisation of skeletal muscle myosin heavy chain in D5 myotubes shows improved organisation and better alignment of sarcomeres in cells on soft surfaces compared to hard surfaces (Fig. 5c).

**Figure 6:**
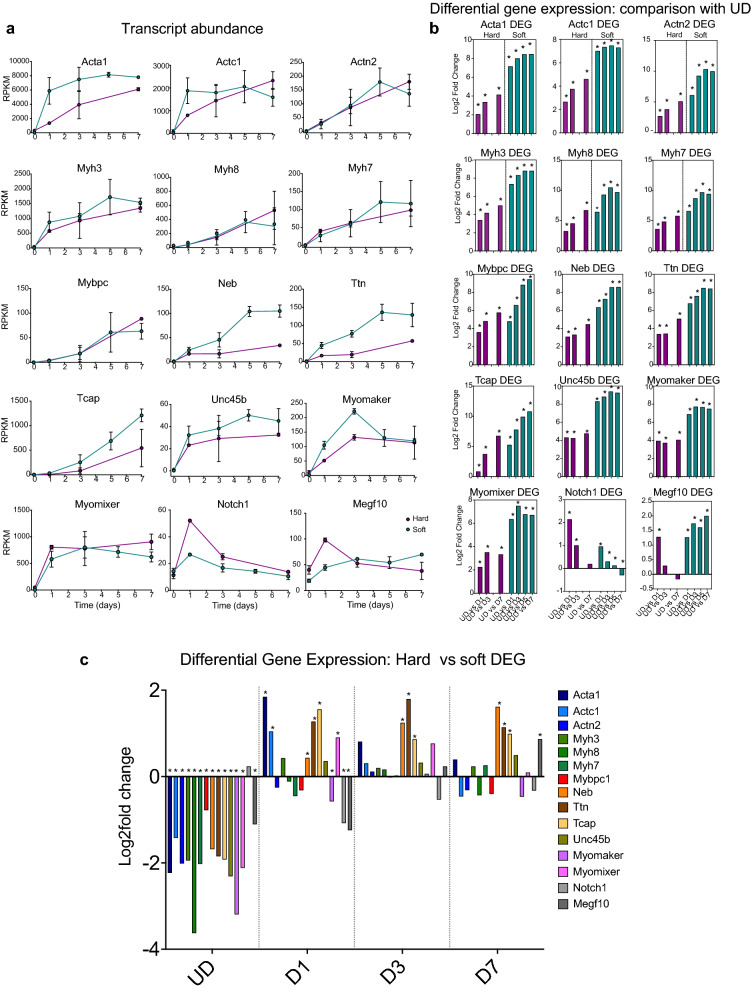
Expression of sarcomeric genes on hard or soft surfaces in C1F cells. (**a**) RPKM plots for hard (magenta) and soft (green) surfaces over time. Day 0 (D0) represents undifferentiated cells (UD). Days 1–7 represent the time course of differentiation. Error bars show the standard deviation (S.D.). (**b**) Differential gene analysis (DEG) that compares expression for each gene on over time, comparing UD with F1 etc. for cells cultured on hard and on soft surfaces. *Indicates significant change in log_2_fold expression (p_adj_ value < 0.05). **c:** DEG comparing expression of each gene for cells cultured on hard versus soft surfaces, at each time point. *Indicates significant change in log_2_fold expression (p_adj_ value < 0.05).

Next, we further analysed the RNA-Seq data to determine the general trend for expression of a selection of sarcomeric proteins, given the improved myofibrillogenesis on soft surfaces shown here and in agreement with previous work using C2C12 cells^11^. We focussed on key structural proteins, such as skeletal (Acta1) and cardiac actin (Actc1), actinin-2 (Actn2: found in the Z-disc), myosin heavy chain genes (Myh3—embryonic, Myh8—perinatal, and Myh7—slow/β-cardiac), titin (Ttn), nebulin (Neb), t-cap, myosin binding protein-C (Mybpc) and Unc45b (Unc45 myosin chaperone b), a specific striated muscle chaperone, important for the folding of skeletal myosin heavy chain^19^. The mRNA expression levels for all of these genes generally increased as the cells differentiated, consistent with the myogenic nature of the C1F clone (Fig. 6a). A differential gene analysis that independently compared expression at D1-D7 with UD showed that expression of all of these genes increased significantly from D1-D7 compared to UD on both surfaces (Fig. 6b). However, changes were more marked for cells cultured on soft surfaces (Fig. 6a,b).

We then performed a differential gene expression analysis that independently compared mRNA expression levels for individual sarcomeric genes between cells cultured on hard and soft surfaces at each time point (Fig. 6c). We found that levels of all of the sarcomeric genes were significantly higher on hard than on soft surfaces in undifferentiated cells (UD), suggesting that growth on soft surfaces might tend to inhibit differentiation. This difference in expression quickly reversed on day 1 for most of these genes, with levels significantly higher in cells cultured on soft surfaces, concomitant with the increase in myogenin levels (Figs. 3, 4b) as cells switch to differentiation.

In addition to the sarcomeric genes, we investigated the expression pattern for two key genes that encode membrane proteins myomixer (Gm7325) and myomerger (Tmem8c). These two genes are important in myoblast fusion and are required to form multinucleated muscle myotubes^20^. The transcript abundance of both genes increased at D1 on both hard and soft surfaces, compared to undifferentiated cells (Fig. 6a). A differential gene analysis independently comparing D1-D7 levels with those of UD showed that levels were increased significantly from D1-D7 compared to UD on both surfaces (Fig. 6b). A differential gene analysis to independently compare expression levels for specific genes on hard vs soft surfaces at each time point showed that myomixer expression is decreased significantly at D1 on hard compared to soft surfaces (Fig. 6c), potentially helping to explain the reduced ability of C1F cells to fuse on hard surfaces.

Notch1 signalling is also known to be important in muscle myogenesis. Notch1 is upregulated when satellite cells are activated and promotes proliferation^21^. Overexpression of Notch1 can inhibit myogenesis^22^. We found that mRNA levels of Notch1 increased between UD and D1 of differentiation on both hard and soft surfaces (Fig. 6a). A differential gene expression analysis that compared levels at D1 with UD showed that this increase was significant (Fig. 6b). However, the level of Notch1 in cells cultured on hard surfaces at D1 is significantly higher than that for cells cultured on soft surfaces (Fig. 6c). This is unexpected because Notch1 expression should decrease at D1, as the time cells cease proliferating and start differentiating.

MEGF10 (multiple EGF like domains 10) has been suggested to interact with Notch1 and to be significantly downregulated when C2C12 cells are induced to differentiate by serum withdrawal^23^. We found that mRNA levels of MEGF10 increased significantly between undifferentiated cells and D1 on both soft and hard surfaces (Fig. 6b). However, on soft surfaces, MEGF10 levels remained significantly elevated at D3-7 compared to UD cells, while on hard surfaces, MEGF10 levels did not (Fig. 6b). On hard surfaces, MEGF10 levels are increased significantly for cells cultured on hard surfaces at D1 (Fig. 6c) but on soft surfaces, increased significantly at D7. These expression patterns argue against the role of MEGF10 in promoting proliferation in combination with Notch1.

Overall, this analysis of gene expression demonstrates that Pax3 positive C1F cells express a range of markers for myogenic differentiation, with a pattern consistent with differentiation of myoblasts into multinucleated myotubes.

### Differential gene expression analysis of collagen genes

The WebGestalt analysis (Fig. 2e–h) showed that growing cells on hard surfaces increased gene expression for a range of extracellular matrix (ECM) and associated proteins, as well as the receptors that bind these proteins. This raised the possibility that cells cultured on hard surfaces attempt to form their own niche, through expression of ECM proteins, to enable them to differentiate and could help to explain the delay in expression of myogenic genes.

**Figure 7:**
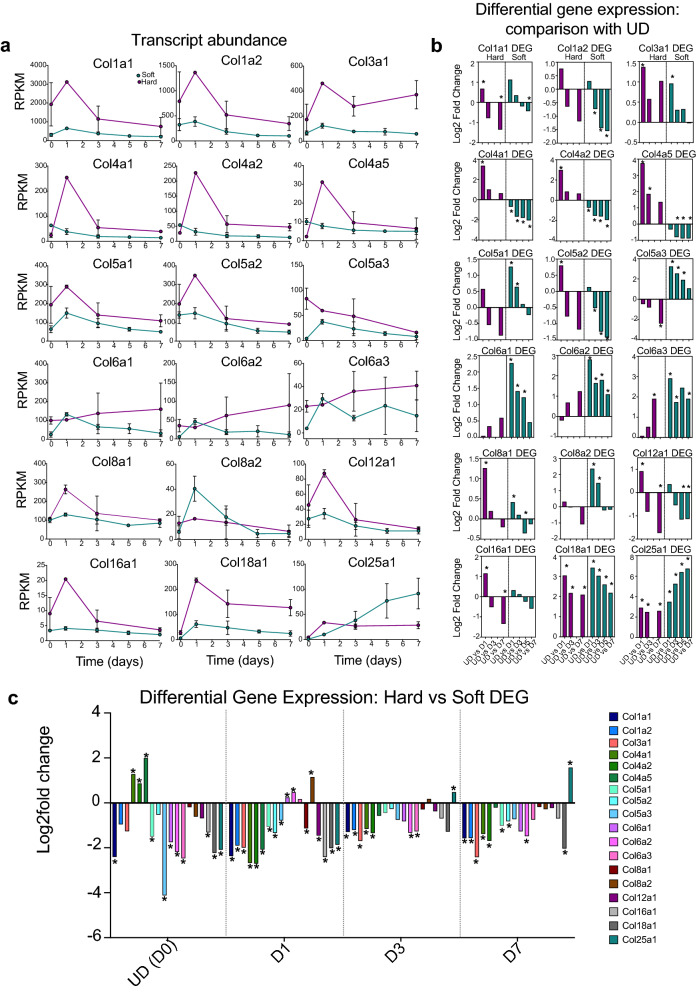
Expression of collagen genes on hard or soft surfaces in C1F cells. (**a**) RPKM plots for hard (magenta) and soft (green) surfaces for genes of interest, over time. Day 0 (D0) represents undifferentiated cells (UD). Days 1–7 represent the time course of differentiation. Error bars show the standard deviation (S.D.). (**b**) DEG that compares expression for each gene over time, comparing UD with F1 etc. for cells cultured on hard and on soft surfaces. *Indicates significant change in log_2_fold expression (p_adj_ value < 0.05). (**c**) DEG comparing expression of each gene for cells cultured on hard versus soft surfaces, at each time point. *Indicates significant change in log_2_fold expression (p_adj_ value < 0.05).

To explore these results further, we analysed the levels of collagen (Col) mRNA, for genes found in the ECM structural constituent category (GO:0005201) between hard and soft surfaces at each time point. This analysis focused on genes with the highest RPKM values and known to be associated with skeletal muscle ECM^24^. Almost all the Col genes analysed showed a peak in mRNA levels in cells cultured on hard surfaces at D1 (Fig. 7a) and levels were generally higher for cell cultured on hard than on soft surfaces from D1-7 (Fig. 7a). A differential gene expression analysis that independently compared levels at D1-7 with UD showed similar trends for most genes on both hard and on soft surfaces (Col1a1 and a2, Col3a1, Col5a1 and 2, both Col8 genes, Col12a1, Col16a1 and Col18a1: Fig. 7b). A differential expression analysis that independently compared expression levels for each gene between cells cultured on hard and soft surfaces at each time point, showed levels of collagen in cells cultured on hard surfaces were generally higher than those cultured on soft surfaces at every time point (Fig. 7c).

While the general trend in differential expression of collagen genes was similar, some differences were observed. In particular, the mRNA expression profiles of Col4 genes (Collagen IV: Col4A1, 4A2 and 4A5) were different between cells cultured on soft and hard surfaces. On hard surfaces, Col4a1, a2 and a5 levels increased at day 1 compared to UD (Fig. 7a) and a differential gene expression analysis showed that levels between UD and D1 were significantly higher for all three (Fig. 7b). In contrast, on soft surfaces, Col4a1, a2 and a5 levels decreased at D1 compared to UD cells, and this decrease was significant for Col4a1 and Col4a2 (Fig. 7b). A direct comparison between each of the Col4 genes in UD cells showed that Col4a1, a2 and a5 levels were significantly lower on hard compared to soft surfaces (Fig. 7c). This trend reversed at D1-7. Also of note is that Col8a2 mRNA levels increased between UD and D1 on soft but not on hard surfaces (Fig. 7b), the reverse of the general trend. Finally, levels for Col25a1 continued to increase from D1 to D7 compared to UD on soft surfaces but remained similar on hard surfaces (Fig. 7b).

Collagen IV is part of the basement membrane, a thin sheath of connective material that surrounds skeletal muscle, and other cell types^25^. A heterotrimer composed of two α1 (Col4a) and one α2 molecules is found in many types of basement membranes, including that of muscle^26^. The increased expression of Col4 genes in cells cultured on hard surfaces at day 1 of differentiation suggests that the hard surface promotes basal lamina formation more strongly than the soft surface, possibly to counteract the hardness of the culture surface. The reasons for differences in expression profiles for Col8a1 and Col25a1 are unclear.

### Differential gene expression analysis of laminin, nidogen and integrin genes

**Figure 8:**
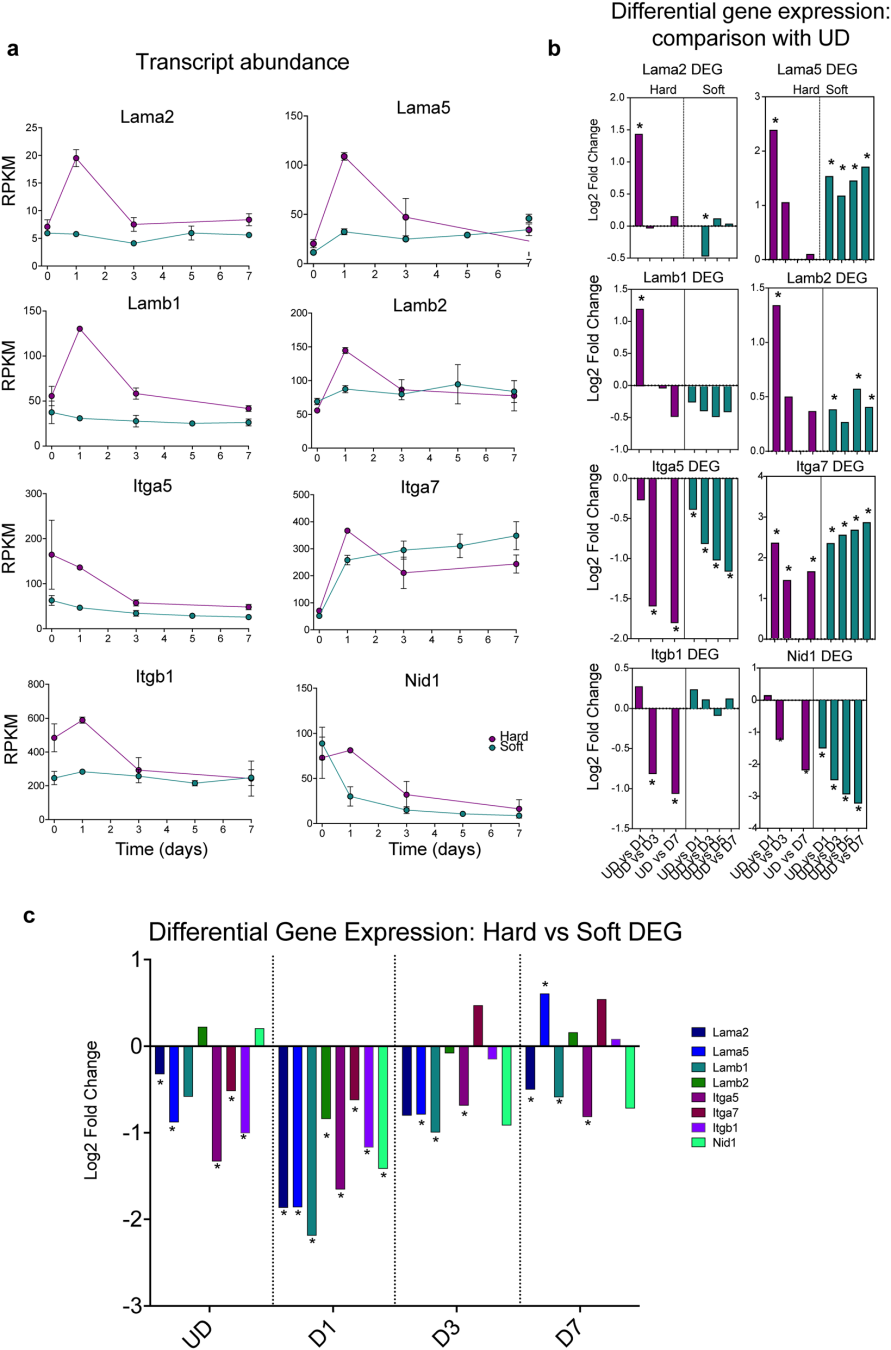
Expression of laminin, integrin and Nid1 (nidogen) genes on hard or soft surfaces in C1F cells. (**a**) RPKM plots for hard (magenta) and soft (green) surfaces for each of the genes of interest, over time. Day 0 (D0) represents undifferentiated cells (UD). Days 1–7 represent the time course of differentiation. Error bars show the standard deviation (S.D.). (**b**) DEG that compares expression for each gene over time, comparing UD with D1 etc. for cells cultured on hard and on soft surfaces. *Indicates significant change in log_2_fold expression (p_adj_ value < 0.05). (**c**) DEG comparing expression of each gene for cells cultured on hard versus soft surfaces, at each time point. *Indicates significant change in log_2_fold expression (p_adj_ value < 0.05).

We performed similar analyses for laminin and nidogen, two proteins also found in basal lamina alongside collagen IV. We found that mRNA levels for laminins (Lama2, Lama5, Lamb1, Lamb2) were much increased at D1 on hard compared to soft surfaces (Fig. 8a). The increase in mRNA level for all of these laminins was significant for cells cultured on hard surfaces, when comparing D1 to UD (Fig. 8b). A direct comparison of levels for the laminin genes showed that levels for all 4 genes were generally higher on hard surfaces compared to soft surfaces at each time point, but most noticeably at D1 (Fig. 8c). Nidogen (Nid1) levels were also higher on hard than on soft surfaces at day 1 (Fig. 8a) and differential gene expression analysis showed that levels were significantly higher on hard than on soft surfaces at this time point (Fig. 8c). This lends support to the idea that differentiation on a hard surface promotes basal lamina formation.

To pursue this idea further, we analysed the expression patterns of three integrin genes: integrin α5 (Itga5), α7 (Itga7) and β1 (Itgb1). Integrins are membrane proteins that bind ECM proteins. Integrin α5, β1, binds to fibronectin (FN1), and its levels have been reported to decrease as myoblast fusion and differentiation progress^27^. Integrin α7, β1 is the main laminin receptor on myoblasts and myotubes and has been reported to be important for myoblast fusion^28^. All three of these genes showed the highest mRNA levels (as judged by RPKM).

Overall, the mRNA levels for all three integrins were higher in cells cultured on hard surfaces compared to cells cultured on soft surfaces at D1 (Fig. 8c). On both surfaces, Itga5 levels decrease from D1-D7 compared to UD and levels of Itga7 increase as expected (Fig. 8b). However, on hard surfaces, levels of Itgb1 decrease significantly at D3 and D7 compared to UD (Fig. 8b), whereas on soft surfaces there is no significant change in Itgb1. This is mainly because Itgb1 levels are higher at UD and D1 on hard compared to soft surfaces, and at subsequent time points, the levels are more similar (Fig. 8a). The higher levels of integrins at D1 match the higher levels of ECM proteins on hard surfaces.

## Discussion

These data show that a clonal cell line of myoblasts, C1F, derived from the H2k^b^-tsA58 immortomouse predominantly expresses Pax3 and not Pax7 and yet follows the same differentiation pathway observed for Pax7 expressing cells^15^. Our findings thus support the evidence that despite the differences in roles of these two genes, Pax3 and Pax7 are capable of triggering a myogenic programme that follows the same transcriptional pattern leading to muscle maturation^29^.

The C1F cells differentiate better on softer (~ 12 kPa) surfaces than on hard surfaces (plastic or glass), consistent with previous observations for the C2C12 cell line^30^. The key difference between differentiation on soft and hard surfaces appears to be an earlier higher expression of two myogenic regulatory factors, MyoD and myogenin, which indicate that cells begin to differentiate earlier on softer surfaces. The increase in MyoD agrees with previous reports of a precocious rise in MyoD on soft surfaces, alongside overall greater expression by cells^31^. Since MyoD activation is necessary for the specification of terminal differentiation and triggers myogenin activation, the entire myogenic process is expedited. Not surprisingly then, we also find an earlier peak in the rise of myogenin on softer surfaces. Our results are also consistent with a previous RNA-Seq analysis for C2C12 cells that showed a greater increase in expression of genes related to sarcomere formation and muscle maturation on micropatterned soft surfaces than non-micropatterned, compared to hard plastic^31^. Interestingly, expression of sarcomeric genes appeared to be supressed in proliferating cells on soft surfaces, compared to those on hard surfaces. This may be consistent with the finding that substrate stiffness can affect satellite cell self-renewal in vitro^32^.

The PCA analysis showed that mRNA levels for cells grown on soft surfaces were clustered separately to cells grown on hard surfaces at each time point. A volcano plot for upregulated and downregulated genes at each time point (UD-D7) demonstrated that the change in gene expression was greatest at D1, where the cells are switching from proliferation to differentiation. A WebGestalt analysis of genes upregulated in cell culture on hard surfaces, compared to soft surfaces, suggested that genes coding for extracellular matrix proteins were highly enriched in cells cultured on hard surfaces. This was supported by further analysis of the mRNA levels for specific ECM genes and integrins. These results suggest that cells cultured on hard surfaces respond by upregulating expression of ECM genes, possibly in an attempt to manipulate their ECM to facilitate differentiation, which then occurs, but at a slower rate than on soft surfaces.

Overall, the RNA-Seq data presented here provides a significant improvement to our current understanding of muscle differentiation. It will be a valuable resource for interrogating changes to gene expression during differentiation of cultured Pax3 positive myoblasts in comparison to myoblasts expressing Pax7. It will also be valuable in improving our understanding into how culturing myoblasts on hard versus soft surfaces affects gene expression and myogenic differentiation.

## Materials and methods

### Preparation of PDMS (polydimethylsiloxane) surfaces

Synthetic culture surfaces were fabricated using Sylgard 184 (Dow Corning, US) elastomer and curing agent. When combined, the elastomer and curing agent form a silicone-based organic polymer known as polydimethylsiloxane (PDMS). A curing agent to elastomer ratio of 1:50 was used to achieve an elastic modulus of about 12 kPa (as reported in supplemental information in ref^33^). Using a w/w ratio, components were weighed into separate glass beakers and autoclaved. Elastomer and curing agent were then mixed together thoroughly with a sterile metal spatula before transfer to a 15 ml falcon tube. The PDMS mixture was centrifuged at 1000×*g* for 2 min to degas, and a snipped pipette tip was then used to dispense 1 ml of the mixture onto the surface of a 60 mm diameter Nunc petri dish. The PDMS was carefully smoothed across the entirety of the surface with a sterile metal spatula and left to set at room temperature for 48 h.

To improve the adherence, fusion and differentiation of cells cultured on the PDMS surfaces, collagen was crosslinked onto the surface^34^. Collagen solution was prepared by mixing 0.1% [w/v] solution of type 1 collagen from calf skin (Sigma) diluted 1 in 10 with sterile water. Sulfo-SANPAH (N-sulphosuccinimidyl-6-(4′-azido-2′-nitrophenylamino) hexanoate ), a hetero-bifunctional crosslinking agent was prepared as a 0.5 mg/ml solution with 50 mM HEPES buffer at pH 8.5. 3 ml Sulfo-SANPAH solution was added to each coated petri dish, to cover the surface. Dishes were then irradiated with a UV source for 8 min to activate the crosslinker. After exposure, spent Sulfo-SANPAH was aspirated, and wells washed with HEPES buffered solution. These steps were then repeated a second time. Next, 3 ml collagen solution was then applied to each surface and allowed to adhere for 4 h at room temperature, before being washed 3 times with PBS. As a comparison to the soft surfaces, non-PDMS coated standard petri dishes were used, coated with the collagen solution for 4 h at room temperature.

### Growth, proliferation and isolation of RNA from cultured cells

To determine levels of gene expression on standard versus PDMS surfaces, RNA was isolated from C1F cells at different stages of differentiation. We isolated C1F myoblasts from the hindlimb muscles of neonatal (1 day old) H2k^b^-tsA58 immortomice^8^ and have already shown them to differentiate into myotubes as previously reported^10^. C1F myoblasts, at passage 3, were recovered from storage, and proliferated in medium composed of 1 × DMEM (Dulbecco’s Modified Eagle Medium) with GlutaMAX supplemented with 20% heat inactivated foetal bovine serum (FBS, Gibco), 2% chick embryo extract (CEE, E.G.G. Technologies) and 1% (v/v) Penicillin/Streptomycin (P/S, Gibco), and 20 U/ml gamma interferon (γFN, Life Technologies)^8^, at 33 °C with 10% CO_2_. For the RNA-Seq experiments, cells were seeded onto standard or PDMS coated 60 mm Nunc petri dishes at a density of 2 × 10^5^ cells/ml and incubated for 24 h before harvesting (UD). To differentiate the cells, the growth medium was exchanged for differentiation medium, composed of 1X DMEM + GlutaMAX (Gibco) supplemented with 4% horse serum (Gibco), 1% CEE and 1% (v/v) P/S, and lacking γFN, the incubation temperature was raised to 37 °C, and CO_2_ levels reduced to 5%. Cells were harvested at days 1, 3, and 7 of differentiation. Cells on the softer surfaces were additionally harvested at day 5 of differentiation. To harvest the cells, the medium was removed, and cells were lysed directly with TRIzol (Invitrogen) reagent, by adding 1 ml TRIzol to each dish, and using a cell scraper to remove the adherent cells into solution. The resulting cell lysate was mixed thoroughly by pipette, placed into labelled Eppendorf tubes and mixed further by vortex. The lysed cell suspensions were stored at − 80 °C until they were sent for processing.

### Sample processing

Processing of C1F samples was performed by the Translational Genomics Unit, St James’s University Hospital, using their standard genomics workflow. Briefly, RNA samples were treated with a TURBO DNA-freeKit (Ambion Inc.) using conditions recommended by the manufacturers, and then cleaned with an RNA Clean & Concentrator™-5 spin column (Zymo Research Corp.) RNA was tested for quality and yield using a NanoDrop 1000 spectrophotometer and an Agilent 2100 Bioanalyzer.

To minimize bubble PCR artefacts, 100 ng of purified total RNA was used in library preparation, following the TruSeq Illumina protocol. In brief, RNA was polyA-selected, chemically fragmented to about 200 nt in size, and cDNA synthesized using random hexamer primers. Each individual library received a unique Illumina barcode. RNA-Seq was performed on an Illumina HiSeq 3000 instrument using 151 bp paired-end reads.

The quality of paired-end FASTQ files was checked through FastQC (https://www.bioinformatics.babraham.ac.uk/projects/fastqc/). Cutadapt^35^ was used to trim off the adapter sequences and reads were trimmed or filtered according to the quality scores with PRINSEQ^36^. The high-quality reads were mapped onto the mouse reference genome (mm10) using STAR^37^ and only uniquely-mapped reads were kept for the downstream analysis. The alignments were further processed using SAMtools^38^ in terms of manipulation and conversion of BAM files. Read summarisation was conducted and read counts for the genes were generated using featureCounts function of Rsubread package^39^. Differential expression analysis for compared two sample groups and the likelihood ratio test (LRT) for time-series experiments were done using DESeq2 package^40^. degPatterns function from DEGreport package was chosen to characterise the patterns of gene expression and cluster genes based on gene expression profiles for 5478 genes (for hard samples) and 8042 genes (for soft samples) that were differentially expressed across time points based on p_adj_ < 0.001 from Likelihood ratio test (LRT) (https://www.bioconductor.org/packages/release/bioc/html/DEGreport.html).

WebGestalt (WEB-based Gene SeT AnaLysis Toolkit^41^) was used for gene set overrepresentation analyses (ORA) under the significance level of FDR < 0.05 (using geneontology and molecular function (non-redundant) genesets). Values of RPKM (Reads Per Kilobase of transcript per Million mapped reads) were used to compare expression levels for specific genes of interest. Final graphs were generated using Prism (GraphPad). RPKM values are shown as mean ± S.D.

### Immunostaining and Fusion Index calculation

To compare fusion into myotubes, C1F cells were cultured on hard (glass) and soft (PDMS) cell culture surfaces, prepared as described above, except that glass coverslips were used instead of petri dishes. For hard surfaces, 50 µl of 0.01% gelatin in sterile water was added to 13 mm diameter round glass coverslips and allowed to set at 37 °C. Excess gelatin was aspirated and the glass surfaces were coated with 0.1% collagen solution. For soft surfaces, coverslips were coated with 50 µl prepared PDMS as described above, spread carefully with a sterile metal spatula, left to set for 48 h, treated with sulfo-SANPAH (sulfosuccinimidyl 6-(4'-azido-2'-nitrophenylamino) hexanoate) and then coated with 0.1% collagen solution.

C1F myoblasts at passage 3 were seeded onto prepared coverslips at a density of 2 × 10^5^ cells/ml. For analysis, cells were fixed using 4% paraformaldehyde in phosphate-buffered saline (PBS) at UD, D1, D3 and D7, washed 3 × with PBS, and then permeabilised with 0.1% Triton X-100 diluted in PBS containing 1% bovine serum albumin (BSA) (Thermo Fisher Scientific), for 5 min. The cells were stained using a primary antibody (A4.1025: which recognises all skeletal muscle myosin heavy chain isoforms^42^). The antibody was diluted 1 in 10 in wash buffer (PBS containing 1% BSA as a blocking agent) together with DAPI (4′6-diaminidino-2-phenylindole) to stain DNA (1/500 dilution), applied to coverslips for 1 h, then removed and coverslips washed 5 times with wash buffer. The secondary antibody (Alexa-Fluor 488 anti-mouse (Thermofisher), diluted 1 in 400, was applied to the coverslips for a further 1 h. The secondary antibody was then removed, coverslips were washed 5 times with wash buffer and once with PBS, then mounted onto glass slides with Prolong Gold anti-fade mountant (Invitrogen). Slides were left to dry at room temperature in the dark overnight and stored at 4 °C before imaging.

To estimate the fusion index, images of the cells were captured using a Delta Vision Widefield Deconvolution Microscope (Delta Vision, USA) using a 40 × objective lens (N.A. 1.4). Three biological repeats were performed, and cell images were taken from 10 random fields of view for each repeat. The fusion index was determined by dividing the total number of nuclei found within myotubes (identified by their positive staining for skeletal myosin by the total number of nuclei in the field of view. Values were plotted and compared using Prizm (GraphPad) using multiple unpaired t-tests. Cells were additionally imaged using an LSM880 confocal microscope (Zeiss) and 40 × objective lens (N.A 1.4) to image myoblast differentiation in more detail.

To investigate expression of transcription factors, cultures at each time point (UD, D1, D5, D7) were fixed and stained for Pax3 (Developmental studies hybridoma bank (DSHB): diluted 1:20), Pax7 (DSHB, diluted 1:20), MyoD (diluted 1:100, Invitrogen, MA1-41017) and myogenin (diluted 1:50, Invitrogen: MA511486) and co-stained using DAPI, using a similar procedure. To quantify expression, three biological repeats were performed for each time point, on each type of surface, and cell images were taken from 10 random fields of view for each repeat. Numbers of nuclei positively stained for each transcription factor, in each field of view were counted. Values were plotted and compared using Prizm (Graphpad) using multiple unpaired t-tests.

## Supplementary Information

Supplementary Figure S1.

## Data Availability

RNA-Seq datasets have been deposited on Sequence Read Archive (SRA) with accession number: PRJNA682314.
