# Supplementary material for: RNA-Seq analysis of a Pax3-expressing myoblast clone in-vitro and effect of culture surface stiffness on differentiation
Source: Sci Rep. 2022 Feb 18;12:2841. doi: 10.1038/s41598-022-06795-3 (PMC8857316; doi:10.1038/s41598-022-06795-3)
Supplement: Supplementary file 1 — Supplementary Figure S1. [file 41598_2022_6795_MOESM1_ESM.docx]

Supplementary Information

**RNASeq analysis of a Pax3-expressing myoblast clone *in-vitro* and effect of culture surface stiffness on differentiation.**

Louise Richardson^1^, Dapeng Wang^2,3^, Ruth Hughes^1^, Colin A Johnson^4^ and Michelle Peckham^1*^

^1^School of Molecular and Cellular Biology, University of Leeds, UK

^2^LeedsOmics, University of Leeds, Leeds, LS2 9JT, United Kingdom,

^3^Wellcome Centre for Human Genetics, University of Oxford, Oxford, OX3 7BN, United Kingdom,

^4^Leeds Institute of Medical Research, Faculty of Medicine and Health, University of Leeds, Leeds, UK

Supplemental Fig. S1





Supplemental Figure S1: Pattern analysis of RNA expression in cells on soft and hard surfaces. The analysis was performed using DegPatterns (see methods).
